# S153. WHERE IS THE ABNORMAL BRAIN ACTIVITY IN FIRST EPISODE PSYCHOSIS?

**DOI:** 10.1093/schbul/sby018.940

**Published:** 2018-04-01

**Authors:** Soldevila-Matias Pau, González-Vivas Carlos, García-Martí Gracián, Soprano-Ros Olga, Martí-Bonmatí Luis, Crespo-Facorro Benedicto, Sanjuán Julio

**Affiliations:** 1 Research Institute of Clinic University Hospital of Valencia (INCLIVA); 2 Clinic University Hospital; 3 Center for Networking Biomedical Research in Mental Health (CIBERSAM), Quirónsalud Hospital; 4 La Ribera University Hospital; 5 Quirónsalud Hospital; 6 Center for Networking Biomedical Research in Mental Health (CIBERSAM), University Hospital Marqués de Valdecilla, IFIMAV, University of Cantabria; 7 Research Institute of Clinic University Hospital of Valencia (INCLIVA), Center for Networking Biomedical Research in Mental Health (CIBERSAM), Clinic University Hospital, University of Valencia School of Medicine

## Abstract

**Background:**

Recent review about functional magnetic resonance imaging (fMRI) in first episode psychosis (FEP) concluded that there is an abnormal connectivity involving the frontal temporal pathway similar to found in chronic schizophrenia (Mwansisya et al., 2017). Besides, thalamic circuits were also altered in chronic schizophrenia patients (Li et al., 2017). The present work gives a wider review of studies using functional magnetic resonance imaging techniques (fMRI) on first-episode psychotic patients, specifically focus on the main areas involved.

**Methods:**

The review was made in accordance with the PRISMA guidelines (Moher et al., 2009). For each study, the following factors were extracted: anatomical location of the main finding and type of functional abnormality (hypo and hyperactivation). A total of 3 different databases (Pubmed, Web of Knowledge, PsycInfo) were reviewed. Thirty-five of 643 (from 2000 to 31st October 2017) neuroimaging papers were analyzed.

**Results:**

We found that the dorsolateral prefrontal cortex (DLPFC) showed 52% of activity abnormalities (55% was hypoactivation). Temporal lobe showed 51% of functional activity altered (61% was hypoactivity). The ventrolateral prefrontal cortex (VLPFC) presented 28% of aberrant fMRI (75% was hyperactivity). Thalamus presented alteration activity with 20% (71% was hypoactivity). The Cingulate was also altered during activation with 20% of patients (85% was hypoactivity). Finally, functional alterations of the Amygdala were present in 14% of the selected patients (80% was hypoactivation).

**Discussion:**

This larger review suggests that there are several possible areas apart from fronto temporal pathways (Mwansisya et al., 2017), that have to be taken into account at the early course of psychosis, such as lymbic system, thalamo-cortical networks and cingulate. These functional activation abnormalities seem to be different to the reported in the previous review. The different results seem to be clearly influenced by the kind of paradigm. Moreover, our finding is not in concordance with the suggestion that thalamic alterations became only prominent at the chronic phase of psychosis (Li et al., 2017).

**References:**

1. Moher, D et al., Preferred reporting items for systematic reviews and meta-analyses: The Prisma statement. PLoS Med, 2009, 6, 1000097.

2. Mwansisya, T et al., Task and resting-state fMRi studies in first-episode schizophrenia: A systematic review. Schizophrenia Research article in press, 2017.

3. Li, T et al., Brain-wide analysis of functional connectivity in first episode and chronic stages of schizophrenia: Schizophrenia Bulletin 2016, 43, 436–448.

